# Ubiquitin-Mediated Effects on Oncogenesis during EBV and KSHV Infection

**DOI:** 10.3390/v16101523

**Published:** 2024-09-26

**Authors:** Rachel Mund, Christopher B. Whitehurst

**Affiliations:** Department of Pathology, Microbiology and Immunology, New York Medical College, Valhalla, NY 10595, USA; rmund@student.nymc.edu

**Keywords:** Epstein–Barr Virus, EBV, Kaposi Sarcoma-associated Herpesvirus, KSHV, gammaherpesvirus, ubiquitin, proteasomal degradation, post-translational modification, tumor virus, oncogenesis

## Abstract

The *Herpesviridae* include the Epstein–Barr Virus (EBV) and the Kaposi Sarcoma-associated Herpesvirus (KSHV), both of which are oncogenic gamma-herpesviruses. These viruses manipulate host cellular mechanisms, including through ubiquitin-mediated pathways, to promote viral replication and oncogenesis. Ubiquitin, a regulatory protein which tags substrates for degradation or alters their function, is manipulated by both EBV and KSHV to facilitate viral persistence and cancer development. EBV infects approximately 90% of the global population and is implicated in malignancies including Burkitt lymphoma (BL), Hodgkin lymphoma (HL), post-transplant lymphoproliferative disorder (PTLD), and nasopharyngeal carcinoma. EBV latency proteins, notably LMP1 and EBNA3C, use ubiquitin-mediated mechanisms to inhibit apoptosis, promote cell proliferation, and interfere with DNA repair, contributing to tumorigenesis. EBV’s lytic proteins, including BZLF1 and BPLF1, further disrupt cellular processes to favor oncogenesis. Similarly, KSHV, a causative agent of Kaposi’s Sarcoma and lymphoproliferative disorders, has a latency-associated nuclear antigen (LANA) and other latency proteins that manipulate ubiquitin pathways to degrade tumor suppressors, stabilize oncogenic proteins, and evade immune responses. KSHV’s lytic cycle proteins, such as RTA and Orf64, also use ubiquitin-mediated strategies to impair immune functions and promote oncogenesis. This review explores the ubiquitin-mediated interactions of EBV and KSHV proteins, elucidating their roles in viral oncogenesis. Understanding these mechanisms offers insights into the similarities between the viruses, as well as provoking thought about potential therapeutic targets for herpesvirus-associated cancers.

## 1. Introduction

The *Herpesviridae* are a group of enveloped, double-stranded DNA viruses. The family includes nine viruses that can infect humans, known as Human Herpes Viruses (HHV), which are categorized into alpha (α), beta (β), and gamma (γ) herpesviruses [1]. The α herpesviruses include the following (HHV-1,2,3): Herpes Simplex Virus-1 (HSV-1), Herpes Simplex Virus-2 (HSV-2), and Varicella-Zoster Virus (VZV). β herpesviruses include the following: HHV-5 (Human Cytomegalovirus), 6A, 6B, and 7. The γ-herpesviruses include the following (HHV-4,8): Epstein–Barr Virus (EBV) and Kaposi Sarcoma-associated Herpesvirus (KSHV), respectively. EBV and KSHV establish latency in certain human cells by forming an episomal DNA element within histones [2]. The viral episome remains within the host cell, with restricted latency-associated gene expression, until it is reactivated into the lytic cycle, wherein it will linearize and begin to produce progeny [3].

EBV and KSHV are both tumor viruses, expressing approximately 85 genes that are important for processes including viral latency, reactivation, lytic replication, infectivity, and oncogenesis, which have been extensively studied and reviewed [4,5].

The Epstein–Barr Virus (EBV) was first discovered in 1964 as the first human tumor virus [6]. It infects about 90% of the worldwide population, is the causative agent of infectious mononucleosis, and is a pathogenic driver of Burkitt lymphoma, Hodgkin lymphoma, and nasopharyngeal carcinoma (NPC) [7,8,9]. EBV also causes lethal immunoblastic lymphomas in those with acquired and innate immune disorders [10]. Further, EBV has been implicated in the pathogenesis of multiple sclerosis, although the mechanism has not yet been elucidated [11,12].

The Kaposi Sarcoma Herpesvirus (KSHV) is most prevalent in sub-Saharan Africa, where >90% of adults have been infected. In the Mediterranean, about 20–30% of adults are infected, and in northern Europe, Asia, and the USA, <10% of adults have KSHV. KSHV was first shown to be the causative agent of Kaposi’s Sarcoma in 1994, a disease which previously had unknown etiology [13,14]. KSHV can also cause two lymphoproliferative disorders, including primary effusion lymphoma (PEL) and multicentric castleman disease (MCD) [15,16]. It also has been shown to cause KSHV inflammatory cytokine syndrome, an inflammatory condition [17].

**Figure 1:**
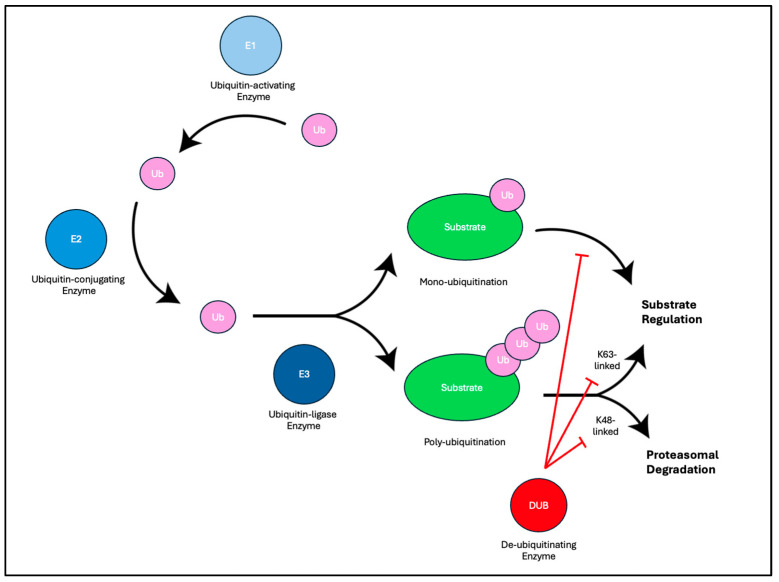
Overview of ubiquitin-conjugating and deubiquitinating enzymes.

These viruses utilize many cellular mechanisms of post-translational modifications (PTMs) to modify protein function and avoid detection within the host cell, including through ubiquitin-controlled substrate regulation and degradation pathways (Figure 1) [4,5]. Ubiquitin is an 8.5 kDa protein found in almost every cell in the body, which commonly serves both regulatory functions, as well as marking substrates for degradation by the proteosome [18]. There are three classes of cellular enzymes (E1, E2, and E3) which are responsible for the ubiquitination of substrates. There are two human E1s, thirty-five E2s, and hundreds of E3s [19,20]. Initially, ubiquitin is activated in an ATP-dependent process by the ubiquitin activating enzyme (E1). The ubiquitin-conjugating enzyme (E2) then promotes the transfer of ubiquitin from the E1 to the active site of the E2 enzyme. Finally, the ubiquitin ligase (E3) performs the final step for creating an isopeptide bond between the lysine residue of the target protein and the C-terminal glycine of ubiquitin [21]. There are exceptions in which amino acids other than lysine residues can be ubiquinated, such as the amino terminal methionine of a protein [22,23]. Protein substrates can be either monoubiquitinated or polyubiquitinated. Ubiquitin contains seven lysine residues (K6, K11, K27, K29, K33, K48, K63), through which polyubiquitin chains are connected [21]. Of these, two in particular are the most studied, Lysine-48 (K-48) and Lysine-63 (K-63), and have been shown to have specific signaling functions. Substrates that are K-48 ubiquitin-tagged are generally associated with that substrate being marked for proteasomal degradation, and K-63 ubiquitin tags tend to have downstream regulatory effects on the tagged substrate [21,24]. Ubiquitin can also be removed from substrates by over 100 cellular deubiquitinating enzymes (DUBs) which further regulate the ubiquitin-mediated pathways [25]. Several viral families, including Picornaviridae, Adenoviridae, Coronaviridae, Bunyaviridae, and Herpesviridae, encode their own deubiquitinating enzymes [26,27,28,29,30,31]. The human herpesvirus encodes a late lytic protein with conserved DUB activity found within the viral tegument [32].

In understanding the mechanism of tumor viruses, it is important to consider the general important drivers of oncogenesis. These include genetic factors, such as mutations, DNA instability, oncogenes, and downregulating tumor suppressor protein expression. Further, cells must allow for uncontrolled cell proliferation, and eventually angiogenesis and metastasis. Finally, successful oncogenesis requires developed mechanisms to evade immune detection. While these oncogenic drivers are all intertwined, they each provide distinct commonalities which can be seen across various cancer types. This review will discuss the ubiquitin-modifying processes occurring during EBV and KSHV infection, which, through the action of viral and cellular ubiquitin ligases and DUBs, ultimately contributes to the oncogenic effects of these viruses.

## 2. Viral Latency Proteins, Ubiquitin Modifications, and Oncogenesis

### 2.1. EBV Latency Proteins

When EBV establishes latency in B-cells, it creates distinct latent gene expression patterns, as follows: types I, II, and III [33]. EBV pathogenic-driven cancers are associated with latency, and different latency patterns have associations with various cancer outcomes. Latency type I shows expression of EBNA1 and is primarily present in Burkitt lymphomas. Latency type II is sometimes divided into IIa and IIb. Latency type IIa shows expression of EBNA1, LMP1, LMP2A, and LMP2B, and is primarily present in Hodgkin lymphomas and nasopharyngeal carcinomas. Latency type IIb shows expression of EBNA1, EBNA2, EBNA3s, EBNALP, and vBcl2, and is primarily present in HIV-associated lymphomas and PTLD cases, as well as almost all early infections [34]. Latency type III shows expression of EBNA1, EBNA2, EBNA3s, EBNALP, LMP1, LMP2A, LMP2B, and vBcl2, and is primarily present in lymphoblastoid cell lines (LCL) and post-transplant lymphoproliferative disease (PTLD) [34]. The EBV latency proteins discussed in this section are summarized in Table 1 below. 

Latent membrane protein 1 (LMP1) is an EBV-encoded protein that has been shown to serve a pathogenic role in many EBV-associated malignancies. LMP1 affects many oncogenic cellular pathways through a variety of mechanisms, including through ubiquitination states. LMP1 has been implicated in apoptosis pathways, including the Nuclear Factor-κB (NF-κB) pathway, important for cellular processes of inflammation, immunity, apoptosis, and growth, as well as with p53, a well-known tumor suppressor [35,36]. Experiments in NPC cell lines have been used to demonstrate that LMP1 has some anti-apoptotic functions in which it can upregulate the NF-κB pathway, thereby enhancing MDM2, an E3 ubiquitin ligase, binding to p53, and leading to increased p53 proteasomal degradation [37,38,39]. Further, MDM2 is overexpressed in many NPC cases, and LMP1 has been shown to increase MDM2 levels and promote stability via K63-linked ubiquitination [40]. LMP1 expression also leads to an upregulation of Bcl-2 expression, another anti-apoptotic factor [38]. When the NF-κB pathway was inhibited, the upregulation of Bcl-2 was diminished, which resulted in the promotion of apoptosis and decreased growth of LCLs, as well as reduced transformation of primary B-cells [37,38,39]. When LMP1 was targeted for increased intracellular degradation via ubiquitination, the LMP1-mediated increase in NF-κB was reversed [41]. Additionally, it has been shown that LMP1 induces the tumor progression locus 2 (TPL2) by activating the inhibitor κB kinase (IKK2)-JNK cascade, likely leading to MAP kinase (MAPK)-mediated oncogenesis [42]. Further, it was shown that TRAF-mediated ubiquitination of the LMP1 membrane-proximal TES1/CTAR1 domain activated MAPK, and TES1 has been shown to be critical in EBV-mediated B-cell transformation [43]. In fact, TRAF1 expression increased LMP1 TES1 domain-mediated activation of the p38, JNK, ERK, and canonical NF-kB pathways [44]. LMP1 alone was shown to increase Uch-L1 expression, which is an endogenous DUB that has been implicated in the development of PEL. Further, while LMP1 alone induces Uch-L1 expression, co-expression of LMP1 and LANA, the KSHV latency-associated nuclear antigen, had an additive effect on Uch-L1 expression [45]. LMP1 also directly interacts with both RIPK1 and RIPK3. LMP1 causes an increased level of K63-polyubiquitinated RIPK1, which downregulates RIPK1 protein expression. LMP1 further inhibits the K63-polyubiquitination of RIPK3, which prevents cellular necroptotic death, promoting cell survival [46]. LMP1 was also shown to decrease levels of Siah-1, an E3 ubiquitin ligase, in B lymphoma cells, activating and stabilizing β-catenin levels in those cells, likely affecting apoptosis [47].

LMP1 also plays a role in angiogenesis. The presence of LMP1 increased proteasomal degradation of prolyl HIF-hydroxylases 1 and 3 via Siah1, decreasing the ubiquitination of HIF1alpha, and ultimately rescuing HIF1alpha from degradation, allowing for increased angiogenesis in nasopharyngeal epithelial cells [48]. LMP1 was also shown to stabilize peroxisome proliferator-activated receptor coactivator-1a (PGC-1α), allowing for anoikis resistance and increased immune evasion in metastatic NPC [49].

Furthermore, the regulation of viral-encoded LMP1 itself is also ubiquitin-mediated. Ribosomal protein s27a (RPS27a) has been shown to directly interact with and stabilize LMP1 by inhibiting its proteasome-mediated ubiquitination [50]. LMP1 also interacts with RNF1, an important component of the linear ubiquitin assembly complex (LUBAC). When RNF1 is downregulated, the functions of LMP1 are hindered, and there is a decrease in cell proliferation [51].

Other EBV latency proteins have also been shown to be involved in ubiquitin-mediated oncogenesis. One such protein is LMP2A, which has been shown to affect B-cell signaling, potentially potentiating oncogenic transformation. LMP2A interacts with and increases Itch, a Nedd4 E3 ligase associated with cell–cell adhesion, and Lyn, a Src family kinase, which regulates B-cell signaling and allows for increased epithelial-mesenchymal transition (EMT)-associated changes [52,53,54,55]. LMP2A has been shown to promote the hyperproliferation of B-cells by enhancing MYC expression and MYC-dependent degradation of the p27^kip1^ tumor suppressor [56]. Another such protein is EBNA1, a latency protein that is vital for latent phase DNA replication and has been shown to compete with p53 for binding with USP7, a DUB which is responsible for regulating the p53 core binding domain [45,57]. Further, EBNA1 interacts with CK2, promoting proteasomal degradation of PML, disrupting DNA repair, and enhancing cell survival [58]. Interestingly, it has been shown that LMP2A can also be ubiquinated at its amino terminus in the absence of lysine amino acids, an example of lysine-independent ubiquitination [22].

There is significant evidence that EBNA3C, a transcription factor vital in EBV LCL transformation, mediates oncogenesis through various ubiquitin-related mechanisms [59]. EBNA3C promotes cell proliferation by stabilizing cyclin D1 and cyclin D2. It stabilizes cyclin D1 by inhibiting its polyubiquitination, inhibiting GSK-3β, which enhances cyclin D1 nuclear localization, and promoting the proteasomal degradation of RASSF1A, a tumor suppressor protein involved in cyclin D1 regulation [60,61,62]. EBNA3C also increases cyclin D2 stability by affecting the proteasomal degradation of Bcl6 [63]. Additionally, EBNA3C promotes cell proliferation by stabilizing Pim-1, inhibiting its proteasomal degradation, and ultimately leading to the downregulation of the cell cycle inhibitor p21/WAF1 [64]. Furthermore, EBNA3C is implicated in DNA instability and obstructs DNA damage repair pathways by downregulating transcription and promoting the proteasomal degradation of H2AX, an essential player in DNA stability and damage repair [65]. Both EBNA3C and EBNA3A have been shown to interact with the USP46/USP12 DUB complex, leading to EBV-associated oncogenesis [66].

**Table 1:** EBV latency ubiquitin-mediated protein interactions contributing to viral oncogenesis.

EBV Latency Protein	Cellular Protein Interactions and Oncogenic Effects
**LMP1:**	Direct Interactions: **TRAF1:** TRAF1 (an E3 ligase) ubiquitinates LMP1 membrane-proximal TES1/CTAR1 domain, activating MAPK, and EBV-mediated B-cell transformation [44]. **RIPK1 and RIPK3:** LMP1 interacts with RIPK1 and RIPK3. It decreases RIPK1 protein expression and increases cell survival through ubiquitin-mediated mechanisms [46]. **RPS27a:** LMP1’s interaction with RPS27a inhibits LMP1’s proteasome-mediated ubiquitination, regulating its function [50]. **RNF1:** LMP1 interacts with RNF1. LMP1 function is inhibited when RNF1 is downregulated, and a decrease in cell proliferation is observed [51]. Indirect Interactions: **NF-κB pathway:** LMP1 upregulates the NF-κB pathway by polyubiquinating TRAF6, leading to MDM2 (an E3 ubiquitin ligase) ubiquitinating p53, and increased p53 proteasomal degradation. There is also an upregulation of Bcl-2 expression [35,37,38,39]. **IKK2-JNK cascade:** LMP1 activates the IKK2-JNK cascade, inducing TPL2- and MAPK-mediated oncogenesis [42]. **Uch-L1 increase:** LMP1 expression increases Uch-L1 (endogenous DUB involved in PEL development) expression [45]. **Angiogenesis pathway:** LMP1 expression causes increased proteasomal degradation of prolyl HIF-hydroxylases 1 and 3, decreasing their ubiquitination of HIF1alpha, and rescues HIF1alpha from degradation, resulting in increased angiogenesis [48]. **PGC-1α pathway:** LMP1 stabilizes PGC-1α via PRMT1, allowing for anoikis resistance and increased immune evasion [49]. **B-catenin pathway:** LMP1 decreases Siah-1 (an E3 ubiquitin ligase) levels and stabilizes β-catenin, preventing apoptosis.
**LMP2A:**	Direct Interactions: **Nedd4 Ligases (Itch, Lyn, AIP4, WWP2, Siah-1):** LMP2A interacts with several Nedd4 E3 ligases, regulating B-cell signaling and allowing for increased epithelial-mesenchymal transition (EMT)-associated changes [52,53,54,55]. Indirect Interactions: **MYC pathway:** LMP2A enhances MYC expression and MYC-dependent degradation of the p27^kip1^ tumor suppressor, causing B-cell hyperproliferation [56].
**EBNA1:**	Direct Interactions: **USP7:** EBNA1 and p53 compete for binding with USP7 (endogenous DUB), which regulates p53’s core binding domain [45,57]. **CK2:** EBNA1 interacts with CK2, increases proteasomal degradation of PML, impairs DNA repair, and increases cell survival [58].
**EBNA3C:**	Direct Interactions: **USP46/USP12:** EBNA3C interacts with the USP46/USP12 DUB complex, leading to EBV-associated oncogenesis [66]. **Cyclin D1:** EBNA3C inhibits the polyubiquitination of cyclin D1 and stabilizes it [60,61,62]. **Bcl6:** EBNA3C modifies the proteasomal degradation Bcl6, causing a secondary increase in cyclin D2 stability [63]. **Pim-1:** EBNA3C stabilizes Pim-1 by inhibiting its proteasomal degradation, leading to downregulation of p21/WAF1 (cell cycle inhibitor) and increased cell proliferation [64]. **H2AX:** EBNA3C downregulates the transcription and promotes the proteasomal degradation of H2AX, leading to increased DNA instability and obstructing DNA damage repair pathways [65]. **SCF^Skp2^:** EBNA3C recruits SCF^Skp2^ (an E3 ubiquitin ligase), a known ubiquitinator of p27, E2F, and c-myc [67].

Footnote: The bolded text refers to the protein being discussed and the underlined text indicates whether the protein interactions are direct or indirect.

### 2.2. KSHV Latency Proteins

KSHV latency is largely controlled by the latency-associated nuclear antigen (LANA). It is of note that the early lytic protein, replication and transcription activator (RTA), is necessary for promoting LANA gene expression and subsequent latency [68]. Other proteins work with LANA to establish and maintain latency. Viral FLICE-inhibitory protein (vFLIP), Kaposin A, and vIRF3 have been shown to be involved in the oncogenesis of latently infected cells [69,70,71,72]. The KSHV latency proteins discussed in this section are summarized in Table 2 below.

LANA plays several roles in viral oncogenesis, many of them ubiquitin-mediated. LANA has been shown to be expressed in all KSHV-associated tumor cells, to interact with the tumor suppressor proteins p53 and pRb, and to be essential for tumorigenesis [73,74,75]. Further, LANA recruits and upregulates the EC(5)S ubiquitin complex, leading to increased p53 proteasomal degradation. This also leads to the dysregulation of Bub1 activity, causing downstream dysregulation of chromosome replication and aneuploidy [76,77]. Moreover, LANA increases Aurora A expression, and Aurora A subsequently enhances the p53-LANA binding affinity [78]. LANA also mediates tumorigenesis through its role in stabilizing beta-catenin, rescuing it from proteasomal degradation, which it achieves through directly interacting with GSK-3 [79]. Further, LANA acts in a GSK-3-independent manner, through which it stabilizes c-Myc by decreasing its ubiquitination and activating ERK1 [80]. LANA also has been shown to interact with FBW7, an E3 ubiquitin ligase, and competitively inhibits its binding to MCL-1, an anti-apoptotic protein [81]. As mentioned above, LANA can increase Uch-L1 activity by interacting with RBP-Jκ, subsequently activating the Uch-L1 promoter [45]. Another oncogenic pathway LANA is implicated in is the NOTCH pathway, as LANA has been shown to directly interact with Sel10, suppressing the ubiquitin-mediated activation of ICN, a protooncogene which is stabilized by the Sel10-mediated ubiquitin-proteasome pathway [82].

Other latency proteins have also been shown to have ubiquitin-mediated roles in the oncogenic process of KSHV. vFLIP, a latency protein that is crucial in the virus’s ability to manipulate the host cell immune system, increased SQSTM1 expression, a ubiquitin-binding protein, and led to the activation of Nrf2, which is usually activated by stress signals. Activated Nrf2 subsequently activates the transcription of cytoprotective genes, which have been implicated in oncogenesis [83]. Further, Kaposin A interacts with and regulates the phosphorylation of the neural-restrictive silencer factor (REST) and the interaction with β-TRCP, an E3 ligase, leading to the over expression of mGluR1, which is important in KSHV-induced cell proliferation [84].

Finally, KSHV-encoded vIRF3 enacts ubiquitin-mediated signaling processes, which ultimately result in anti-apoptotic pathways. vIRF3 is a viral lytic protein that is important in apoptosis, cell cycle, antiviral immunity, and tumorigenesis [72]. vIRF3 interacts with and inhibits p53-mediated apoptosis by blocking p53 phosphorylation at serine residues S15 and S20. vIRF3 destabilizes p53, increasing p53 polyubiquitination and targeting p53 for proteasome-mediated degradation [72].

**Table 2:** KSHV latency ubiquitin-mediated protein interactions contributing to viral oncogenesis.

KSHV Latency Protein	Cellular Protein Interactions and Oncogenic Effects
**LANA:**	Direct Interactions: **p53 and pRb:** LANA interacts with both p53 and pRb. LANA recruits and upregulates the EC(5)S ubiquitin complex, enhancing p53 proteasomal degradation [73,74,75,76,77]. **Bub1:** LANA dysregulates Bub1 activity, leading to downstream problems with chromosome replication and aneuploidy [76,77]. **GSK-3:** LANA interacts with GSK-3, which stabilizes beta-catenin and rescues it from proteasomal degradation, mediating tumorigenesis [79]. **MYC:** LANA stabilizes c-Myc by decreasing its ubiquitination and activating ERK1 [80]. **FBW7:** LANA competitively inhibits FBW7 (an E3 ubiquitin ligase), binding to MCL-1, an anti-apoptotic protein [81]. **RBP-Jκ:** LANA interacts with RBP-Jκ, increasing Uch-L1 activity and activating the Uch-L1 promoter [45]. **Sel10:** LANA interacts with Sel10, suppressing the ubiquitin-mediated activation of ICN. ICN is stabilized by the Sel10-mediated ubiquitin-proteasome pathway of the NOTCH pathway [82]. Indirect Interactions: **p53 pathway:** LANA increases Aurora A expression, enhancing the p53-LANA binding affinity [78].
**vFLIP:**	Indirect Interactions: **Nrf2 pathway:** vFLIP increases SQSTM1 (ubiquitin-binding protein) expression, activating Nrf2 (stress activated protein), and ultimately upregulating the transcription of cytoprotective genes implicated in oncogenesis [83].
**Kaposin A:**	Direct Interactions: **REST and β-TRCP:** Kaposin A regulates the phosphorylation of the neural-restrictive silencer factor (REST) and β-TRCP (an E3 ligase), leading to the overexpression of mGluR1, which is crucial for KSHV-induced cell proliferation [84].
**vIRF3:**	Direct Interactions: **p53:** vIRF3 blocks p53 phosphorylation and promotes its ubiquitin-mediated degradation, preventing p53-mediated apoptosis [72].

Footnote: The bolded text refers to the protein being discussed and the underlined text indicates whether the protein interactions are direct or indirect.

## 3. Viral Lytic Proteins, Ubiquitin Modifications, and Oncogenesis

### 3.1. EBV Lytic Proteins

EBV’s lytic cycle is split into three phases based on temporal gene expression, as follows: “immediate early”, “early”, and “late”. The immediate early and early genes include viral transactivators and genes that are necessary for viral DNA replication, whereas late phase genes usually include the transcription of proteins involved in viral particle formation [85]. It is clear that several EBV lytic proteins that interact with the ubiquitin-proteasome system, such as the immediate early proteins BZLF1 and BRLF1, as well as the late protein BPLF1, play significant roles in oncogenesis. By disrupting normal cellular processes, stabilizing viral and host proteins, and evading immune responses, these viral proteins create a cellular environment that is favorable for cancer development (Table 3).

BZLF1 and BRLF1 are immediate early proteins necessary in initiating the switch from the latent to the lytic phase of the viral life cycle [85]. BZLF1 is involved in ubiquitin-mediated oncogenic functions in the lytic cycle. One example of this is the evidence that the presence of BZLF1 increases the proteasomal degradation of p53, independent of MDM2, leading to increased cell proliferation [86]. Further, humanized mice infected with a BZLF1 KO virus developed less tumors than those with the WT virus [86,87]. BZLF1 serves a similar role, in this respect, to vIRF3, a viral lytic protein in KSHV [88]. In addition, it has been shown that TRIM5α interacts with and promotes the ubiquitination of BRLF1 [89].

In 2005, it was discovered that the Herpesviridae encode a conserved deubiquitinating enzyme [27,90]. BPLF1 (EBV’s DUB) is a late lytic cycle gene and tegument protein with its DUB activity located in the N-terminal region. BPLF1 cleaves both K48- and K63-linked polyubiquitin chains and removes ubiquitin from monoubiquitinated targets. DUB activity is abolished by mutating the active site cysteine [28,91]. Further, BPLF1 is important for infectious virus production, B-cell immortalization, and tumorigenesis [28,92]. It was shown that a BPLF1 knockout virus produces a 90% less infectious virus than a wild-type virus [28,92,93]. Additionally, the well-known hallmark of EBV, its ability to transform B-cells in culture, was reduced and delayed with the BPLF1 KO virus [28,92]. Further, humanized mice infected with the BPLF1 KO virus survived longer and had a lower incidence of splenic tumors [92].

BPLF1 may serve as an important oncogenic factor, as it disrupts DNA repair and interacts with many cellular DNA repair proteins. BPLF1 was shown to remove monoubiquitination from PCNA, an important step in translesion synthesis DNA repair [28,91]. BPLF1 acts by hindering the PCNA recruitment of TLS polymerase, pol eta, to stalled replication forks, thereby preventing cellular DNA repair [91]. Pol eta was found to directly interact with BPLF1 and viral DNA, suggesting that pol eta may aid in the bypass of DNA damage during viral replication [94]. BPLF1 also interacts with the E2/E3 ubiquitin complex (Rad6/18) that ubiquitinates PCNA and upregulates Rad18 protein levels [95]. BPLF1 deubiquitinates Rad6, which ultimately reduces H2B ubiquitination and leads to an overall increase in unresolved double-stranded DNA breaks [95,96]. Additionally, BPLF1 was also shown to have DUB activity on topoisomerase-II, resulting in its stabilization, and promoting cell survival [97]. Interestingly, BPLF1 was shown to have DUB activity on EBV’s virally encoded Ribonucleotide Reductase (RR), possibly affecting its function in dNTP synthesis [28].

BPLF1 also plays important functions in EBVs’ ability to avoid immune detection. BPLF1 interacts with and inhibits TRAF6 activation, thereby suppressing interferon (IFN) production and NFκB activation [98]. BPLF1 has also been shown to have DUB activity on p62, allowing for further inhibition of the innate immune response [99]. Furthermore, BPLF1 interacts with and ubiquitinates STING. A transient interaction with TBK1 was also observed. These interactions lead to increased cGAS-STING and TBK1-induced IFN production, which was shown to be reversed by a catalytically inactive DUB mutant [100]. Additionally, BPLF1 interacts with the E3 ligase TRIM25, promoting TRIM25 auto-ubiquitination, and ultimately reducing RIG-I ubiquitination and its signaling [101].

Other EBV lytic proteins that play important roles in viral immune evasion are BDLF3 and BGLF2. BDLF3 indirectly causes a reduction in the surface expression of both major histocompatibility complex I (MHCI) and major histocompatibility complex II (MHCII) surface expression [102]. BGLF2 directly interacts with TYK2, a key protein in the type I interferon signaling pathway [103]. Additionally, BGLF2 indirectly triggers Cullin 1, an E3 ligase, to attach K-48-linked ubiquitin to STAT2, resulting in its proteasomal degradation [104].

**Table 3:** EBV lytic ubiquitin-mediated protein interactions contributing to viral oncogenesis.

EBV Lytic Protein	Cellular Protein Interactions and Oncogenic Effects
**BZLF1:**	Indirect Interactions: **P53:** BZLF1 increases the proteasomal degradation of p53, independent of MDM2, leading to increased cell proliferation [86].
**BRLF1:**	Direct Interactions: **TRIM5α:** BRLF1 interacts with TRIM5α. TRIM5α promotes ubiquitination of BRLF1 [89].
**BPLF1:**	Direct Interactions: **PCNA:** BPLF1 deubiquitinates and hinders PCNA recruitment of TLS polymerase to stalled replication forks, thereby preventing DNA repair [91]. **Topoisomerase-II:** BPLF1 has DUB activity on topoisomerase-II resulting in its stabilization and promoted cell survival [97]. **Rad6/18:** BPLF1 deubiquitinates Rad6/18, which ultimately reduces H2B ubiquitination and leads to an overall increase in unresolved double-stranded DNA breaks [95,96]. **TRAF6:** BPLF1 interacts with and inhibits TRAF6 activation, suppressing IFN production and NFκB activation, preventing immune detection [98]. **STING and TBK1:** BPLF1 interacts with STING, and likely TBK1 as well, affecting the cGAS-STING- and TBK1-induced IFN production, aiding in immune evasion [100]. **TRIM25:** BPLF1 promotes TRIM25 (an E3 ligase) auto-ubiquitination, reducing RIG-I ubiquitination and its signaling [101]. P62: BPLF1 deubiquitinates p62 [99]. **Viral Ribonucleotide Reductase (RR):** BPLF1 has DUB activity on RR [28].
**BDLF3:**	Indirect Interactions: **MHCI/II:** BDLF3 downregulates the surface expression of MHCI and MHCII [102].
**BGLF2:**	Direct Interactions: **TYK2:** BGLF2 interacts with TYK2 (important in the type I interferon signaling pathway) [103]. Indirect Interactions: **Cullin 1:** BGLF2 causes Cullin 1 (an E3 ligase) to add K-48-linked ubiquitin to STAT2, leading to its proteasomal degradation [104].

Footnote: The bolded text refers to the protein being discussed and the underlined text indicates whether the protein interactions are direct or indirect.

### 3.2. KSHV Lytic Proteins

The lytic cycle of KSHV, similar to EBV, involves the production of new virions and the eventual lysis of the host cell. The lytic cycle is initiated by the expression of immediate early genes, followed by early and late gene expression, leading to viral DNA replication and the assembly of new virions [105]. Many of the viral proteins contribute to viral oncogenesis through ubiquitin-medicated mechanisms (Table 4). The immediate early protein replication and transcription activator (RTA), and the viral late lytic DUB Orf64, are immune evasion regulators [106,107]. Further, viral proteins, including viral Interferon Regulatory Factor 1 (vIRF1), viral G Protein-Coupled Receptor (vGPCR), viral Interferon Regulatory Factor 4 (vIRF4), K3, and K5, are expressed during the lytic cycle and promote proliferation and angiogenesis, further contributing to oncogenesis [108,109,110].

KSHV encodes viral proteins, similar to those in EBV, that aid in immune evasion. Like EBV’s immediate early gene BRLF1, RTA, encoded by the Orf50 gene, is the master regulator of the KSHV lytic cycle. RTA is vital to the viral switch from the latent to lytic cycle, as it is responsible for activating the expression of viral lytic genes [107]. It has been shown that RTA has E3 ligase activity and can impair cellular innate immunity by ubiquitinating Myd88 and causing its increased proteasomal degradation [111]. Further, RTA interacts with and ubiquitinates STAT6, promoting its proteasomal degradation and ultimately increasing the degradation of TRIML2, a tumor suppressor protein [112]. Another analog of an EBV protein is Orf64, the KSHV viral DUB, similar to BPLF1, which is discussed above. Orf64 is a late lytic viral protein which is important for viral replication, virion assembly, and immune modulation. Orf64, like BPLF1, is effective at removing both lysine 48 (K48)- and lysine 63 (K63)-linked ubiquitin chains [106]. Orf64 is important for immune evasion, as it suppresses retinoic acid-inducible gene I (RIG-I)-mediated IFN signaling by deubiquitinating RIG-I, thus preventing its activation [113]. Orf64 was also shown to inhibit P53 in a dose-dependent manner, as well as inhibiting the transactivation of BAX and PIG3, which are mediators of p53 [114].

KSHV has several viral enzymes which promote proliferation and oncogenesis through a ubiquitin-mediated mechanism. One such protein is vIRF1, which is responsible for regulating multiple signaling pathways in KSHV infection and KSHV-mediated oncogenesis. It has been shown that the E3 ubiquitin ligase, Kelch-like 3 (KLHL3), is upregulated by vIRF1, which ultimately results in the ubiquitin-proteasome-mediated downregulation of heterogeneous nuclear ribonuclear protein Q1 (hnRNP Q1). This downregulation of hnRNP Q1 ultimately causes increases in glucose uptake, ATP production, and lactate secretion [110]. vIRF1 acts similarly to EBNA1, in EBV, in which it interacts with USP7, destabilizing p53 [115]. KSHV also encodes the enzyme vIRF4, which also interacts with USP7, a protein which is responsible for regulating the stability of p53 and MDM2. vIRF4 interacts with USP7 specifically through its two derived peptides, vif1 and vif2, which serve as selective USP7 antagonists, leading to the inhibition of its ability to regulate tumor suppression [116].

Further, vGPCR, a viral protein expressed during the lytic cycle, can activate multiple signaling pathways, including those involved in angiogenesis and cell proliferation, contributing to KSHV-associated tumorigenesis [117]. It has been shown that vGPCR can induce transforming growth factor β (TGF-β)-activated kinase 1 (TAK1), ultimately activating the NF-κB pathway, via phosphorylation and lysine 63-linked polyubiquitination of TAK1 [117]. When a TAK1-inactivated kinase mutant (TAK1M) was used, vGPCR-induced NF-κB nuclear translocation and transcription were reduced [117]. vGPCR also induces many mediators, including interleukin 8 (IL-8), Gro1, IκBα, COX-2, cIAP2, and Bcl2 genes. All of these functions were impeded by TAK1M expression [117]. Further, vGPCR interacts with Beclin 2, an autophagy-associated protein. Beclin 2 encourages vGPCR’s endolysosomal degradation, thereby inhibiting vGPCR-driven oncogenic signaling. It was shown that monoallelic loss of Becn2 in mice accelerated vGPCR-induced lesions resembling KSHV [118].

KSHV encodes two viral proteins, K5 and K3, which can act as E3 ligases, which are important in immune evasion, particularly through their role in downregulating MHCI [109]. K5 is regulated by the RING domain and a membrane-proximal lysine in the cytoplasmic domain of BMPR-II. When K5 is ectopically expressed, it can cause BMPR-II ubiquitination and lysosomal degradation. This causes an overall decrease in BMP signaling [119]. K5 has been shown to ubiquitinate VE-cadherin for proteasomal destruction, decreasing endothelial cell (EC) adhesion [108]. K5 also increases the proteasomal degradation of alpha-, beta-, and gamma-catenins in EC’s, resulting in a nonfunctional EC barrier due to a rearranged actin cytoskeleton. Interestingly, K5-expressing cells had increased N-cadherin levels [120]. K5 also ubiquitinates the cellular growth factor-binding receptor tyrosine kinase (RTK), leading to increased endocytosis of RTK, increased cellular metabolism, and increased Akt activation, as well as the duration of Erk1/2 phosphorylation [121]. K3, not K5, is responsible for the downregulation of HLA-C and HLA-E [109]. K3 also significantly reduces PECAM-1 expression through its E3 ligase activity [122]. Both K3 and K5 specifically target IFN-γR1, inducing its ubiquitination and proteasomal degradation, resulting in its surface expression downregulation and decreased cellular activity [123].

**Table 4:** KSHV lytic ubiquitin-mediated protein interactions contributing to viral oncogenesis.

KSHV Lytic Protein	Cellular Protein Interactions and Oncogenic Effects
**RTA:**	Direct Interactions: **Myd88:** RTA ubiquitinates Myd88, increasing its ubiquitin-mediated degradation and impairing cellular innate immunity [111]. **STAT6:** RTA ubiquitinates STAT6, increasing its proteasomal degradation, eventually increasing the degradation of the tumor suppressor TRIML2 [112].
**Orf64:**	Direct Interactions: **RIG-I:** Orf64 has DUB activity on RIG-I, decreasing its activation, allowing for viral immune evasion and an ultimate inhibition of p53 expression [113,114].
**vIRF1:**	Direct Interactions: **USP7:** vIRF1 interacts with USP7, destabilizing p53 [115]. Indirect Interactions: **Increased Metabolism:** Kelch-like 3 (an E3 ubiquitin ligase) is upregulated by vIRF1, causing downregulation of hnRNP Q1, and ultimately increased glucose uptake, ATP production, and lactate secretion [110].
**vIRF4**	Direct Interactions: **USP7:** vIRF4 interacts with USP7 through two derived peptides, vif1 and vif2, which suppress USP7’s regulatory effect on p53 and MDM2 [116].
**vGPCR:**	Direct Interactions: **TAK1:** vGPCR induces TAK1, activating the NF-κB pathway [117]. **Beclin 2:** vGPCR interacts with Beclin 2 (autophagy-associated protein) which increases vGPCR’s endolysosomal degradation, thereby inhibiting vGPCR-driven oncogenic signaling [118].
**K5:**	Direct Interactions: **BMPR-II:** K5 ubiquitinates BMPR-II, leading to increased lysosomal degradation and a decrease in BMP signaling [119]. **VE-cadherin:** K5 ubiquitinates VE-cadherin, targeting it for proteasomal degradation, decreasing endothelial cell (EC) adhesion. K5 upregulates proteasomal degradation of alpha-, beta-, and gamma-catenins in ECs, leading to a nonfunctional EC barrier [120]. **RTK:** K5 ubiquitinates RTK, promoting its endocytosis and enhancing cellular metabolism and cell proliferation pathways [121]. **IFN-γR1:** K5 induces the ubiquitination and proteasomal degradation of IFN-γR1, downregulating its surface expression and decreasing its cellular activity [123].
**K3:**	Direct Interactions: **MHC class I molecules** (HLA-A, -B, -C, and -E): K3 downregulates MHCI molecules [124]. **PECAM:** K3 has E3 ligase activity on PECAM-1, lowering PECAM expression [122]. **IFN-γR1:** K3 promotes the ubiquitination and proteasomal degradation of IFN-γR1, leading to reduced surface expression and diminished cellular activity [123].

Footnote: The bolded text refers to the protein being discussed and the underlined text indicates whether the protein interactions are direct or indirect.

## 4. Conclusions and Discussion

**Figure 2:**
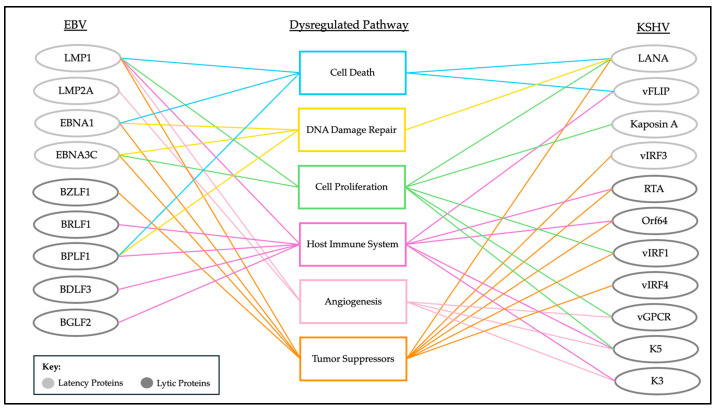
Overview of oncogenic processes disrupted by virally encoded proteins through ubiquitin-mediated mechanisms.

This review explores the ways in which both EBV and KSHV use ubiquitin-mediated mechanisms to enact oncogenesis. It is clear that there is a relationship between viral proteins, the cellular ubiquitin-regulatory pathways they manipulate, and the resulting viral persistence and oncogenic processes. Both EBV and KSHV encode viral proteins that interact with cellular ubiquitin ligases, DUBs, and substrates, influencing critical cellular functions such as apoptosis, immune response evasion, DNA damage repair, angiogenesis, and cell cycle regulation (Figure 2).

EBV latency proteins play pivotal roles in oncogenesis through various ubiquitin-mediated mechanisms. LMP1’s ubiquitin-mediated interactions with the NF-κB pathway, p53, and RIPK1/RIPK3, help account for LMP1’s role in preventing cell death and enhancing oncogenic potential [35,37,38,39,46]. Additionally, the involvement of LMP2A and EBNA3C in B-cell signaling and cell cycle regulation showcases the multifaceted nature of EBV’s oncogenic processes [52,53,54,55,59]. The lytic phase proteins further contribute to EBV-mediated oncogenesis. BZLF1’s promotion of p53 degradation, and BPLF1’s disruption of DNA repair mechanisms, further contribute to tumorigenesis [28,86,91,95,96,97]. The ability of BPLF1 to allow for immune evasion by targeting TRAF6, STING, and TRIM25 through its DUB activity further enforces the importance of ubiquitin-mediated immune evasion processes [98,100,101].

KSHV also utilizes many ubiquitin-mediated processes in viral latency and oncogenesis. LANA’s interactions with p53, pRb, and β-catenin allow for the disruption of tumor suppressor pathways and increased cell proliferation [73,74,75,76,77]. LANA also modulates the NOTCH pathway and the inhibition of Sel10-mediated ubiquitination, allowing for viral interruption of cellular signaling pathways for oncogenic purposes [82]. KSHV’s lytic proteins also play significant ubiquitin-mediated roles in immune evasion processes. RTA’s E3 ligase activity and its ability to degrade Myd88 and STAT6 allows for important viral evasion of innate immunity [111,112]. Orf64’s deubiquitinating activity, which targets RIG-I and p53, allows for both immune evasion and increased cell survival [113,114].

As described above, both EBV and KSHV viral proteins use ubiquitin modification pathways to promote cell survival, proliferation, and immune evasion. While some proteins serve to stabilize cellular proteins, preventing their degradation, others serve as destabilizing forces, disrupting cellular processes through ubiquitin-mediated degradation. These stabilizing and destabilizing forces are particularly interesting when considered in the context of viral oncogenesis. It is clear that these viruses have complex mechanisms to manipulate cellular ubiquitin pathways, allowing them to evade host immune responses, promote cell survival, and drive oncogenic transformation through modulating ubiquitin ligases and DUBs. Both EBV and KSHV encode viral proteins that interact with cellular ubiquitin ligases such as MDM2, TRAFs, and FBW7 [35,37,38,39,44,81,98,100,101]. This leads to a change in the ubiquitination status of critical cellular proteins involved in apoptosis, cell cycle regulation, and DNA repair. It is of note however, that many viral proteins play dual roles in both promoting and inhibiting apoptosis through ubiquitin-mediated mechanisms, emphasizing the complexity of viral pathogenesis.

Considering the viral oncogenic mechanisms of genetics, including DNA repair and gene transcription, uncontrolled cell division and spread, and, finally, immune evasion, it is apparent that EBV and KSHV viruses share a lot of homology in the mechanisms by which they enact their tumor virus functions. Further, given how closely related the viruses are, it is interesting to consider the mechanistic roles their “oncogenes” play in viral oncogenesis, comparing and contrasting how these similar proteins interface with cellular processes. Starting with downregulating tumor suppressor functions, several viral proteins, including LMP1, EBNA1, BZLF1, LANA, vIRF1, vIRF3, vIRF4, and Orf64, act on p53 and the important tumor suppressor protein [35,37,38,39,45,57,72,73,74,75,76,77,86,113,114,115,116]. Further, when reflecting on the proteins associated with proliferation and angiogenesis, LMP1, LMP2A, and vGPCR enact their effects through the MAPK and NF-κB pathways [42,44,52,53,54,55,117]. Finally, when addressing viral mechanisms used for host immune evasion, LMP1, BPLF1, BDLF3, BGLF2, Orf64, RTA, vFLIP, K3, K5, vIRF1, and vIRF3 all act to dysregulate the host immune response to viral dysregulation [49,83,98,100,102,103,113,114,123].

Future research is warranted on the interactions between viral proteins and cellular ubiquitin ligases, which may serve as a novel platform for understanding and treating EBV- and KSHV-associated cancers. Further, this research on the ubiquitin-modifying enzymes involved in viral pathogenesis could reveal biomarkers for the early detection of virus-associated cancers. Both EBV and KSHV exploit ubiquitin-mediated mechanisms to subvert host cellular pathways, facilitating viral persistence and promoting oncogenesis. Due to this, elucidating these ubiquitin-mediated interactions is crucial in the mechanistic understanding of EBV- and KSHV-associated cancers.
